# Isoflavones prevent bone loss following ovariectomy in young adult rats

**DOI:** 10.1186/1749-799X-3-12

**Published:** 2008-03-02

**Authors:** Yang-Hwei Tsuang, Li-Ting Chen, Chang-Jung Chiang, Lien-Chen Wu, Yueh-Feng Chiang, Pei-Yu Chen, Jui-Sheng Sun, Chien-Che Wang

**Affiliations:** 1 Department of Orthopedic Surgery, Taipei City Hospital, Taipei, Taiwan, Republic of China; 2 Institute of Clinical Medicine, National Yang-Ming University, Taipei, Taiwan, Republic of China; 3 Department of Research and Development, Healthbanks Biotechnology Corporation Ltd; Taipei, Taiwan, Republic of China; 4 Department of Orthopedic Surgery, National Taiwan University Hospital, Taipei, Taiwan, Republic of China; 5 Department of Orthopedic Surgery, PoJen General Hospital, Taipei, Taiwan, ROC

## Abstract

Soy protein, a rich source of phytoestrogens, exhibit estrogen-type bioactivity. The purpose of this study was to determine if ingestion of isoflavones before ovariectomy can prevent bone loss following ovariectomy. Twenty-four nulliparous Wistar rats were randomly divided into four groups. In the normal diet groups, a sham operation was performed on Group A, while ovariectomy was performed on Group B. For Groups C and D, all rats were fed with an isoflavone-rich (25 mg/day) diet for one month, then bilateral ovariectomy were performed. In the rats in Group C, a normal diet was begun following the ovariectomy. The rats in Groups D continued to receive the isoflavone-rich diet for two additional months postoperatively. All rats were sacrificed 60 days after surgery. The weight of bone ash of the long bones and whole lumbar spine were determined. A histological study of cancellous bone was done and biochemical indices of skeletal metabolism were performed and analyzed. The markers of bone metabolism exhibited no significant changes. When compared with the sham-operated rats fed a normal diet, the bone mass of ovariectomized rats decreased significantly; pre-ovariectomy ingestion of an isoflavone-rich diet did not prevent bone loss. The bone mass of rats treated with an isoflavone-rich diet for three months was higher than controls two months after ovariectomy.

Dietary isoflavones did not prevent the development of post-ovariectomy bone loss, but long-term ingestion of an isoflavone-rich diet increased the bone mineral contents after ovariectomy in young rats.

## Introduction

Osteoporosis is a complex disease characterized by a reduction in bone mass with associated microarchitectural deterioration and a correspondingly high risk of fractures. It is a serious and costly public health problem in the elderly population [1]. Osteoporotic fractures are an important cause of disability. Osteoporosis and the consequences of compromised bone strength, particularly vertebral and hip fractures, is a significant cause of increased morbidity and even mortality. Hip fractures are associated with a 20% mortality in the year following the fracture [2]. As driven by the aging of the baby boomers and by the increasing longevity, the size of the population aged 50 years or older will increase markedly during the next several decades. Consequently, the cost of managing fractures is substantial and will continue to rise [3]. Thus, the direct, as well as indirect, costs of fractures are expected to increase correspondingly worldwide [4].

Recent epidemiological studies have suggested that the incidence of osteoporosis varies among populations, as the result of the complex interactions of a variety of genetic, geographic, and ethnic factors [5-7]. Although family and twin studies have suggested that there is a strong genetic component which predisposes to osteoporosis, environmental factors like nutrition, mechanical load, and lifestyle are also important [8]. The relative impact of environmental and genetic factors on the predisposition to osteoporosis is still a matter of debate.

Investigation of the role of dietary habits on the development and prevention of postmenopausal osteoporosis has focused primarily on calcium intake and vitamin D repletion. These two factors, albeit important, only account for the development of osteoporosis in part. Indeed, the incidence of osteoporosis in homogeneous populations correlates inversely with calcium intake [9], and it appears that the highest total incidence of fractures is experienced by populations with the highest calcium consumption [10]. Since differences in nutrition between populations can be striking and are not limited to calcium intake, it is possible that other macro- and micro-nutrients may contribute to the different incidence of fractures between populations [11].

Recently, the beneficial effects of various classes of phytoestrogens present in nature were reported [12]. Phytoestrogens are abundant in plants and have received increasing attention as dietary components that can affect several aspects of human health [13]. Phytoestrogens are non-steroidal plant molecules whose structure differs from gonadal hormones, yet have estrogen-type bioactivity [12,14]. The notion that phytoestrogens may affect osteoporosis favorably has emerged recently [15] and is supported by the observations that phytoestrogens are beneficial for manifestations of the postmenopausal state such as hyperlipidemia [16] and hot flushes [17]. Moreover, the synthetic phytoestrogen, ipriflavone, exerts positive effects on the bone mass of postmenopausal women [18] and experimental animals [19]. This beneficial effect has also been reported from human feeding trials [20,21].

Soy protein and flaxseed are the most common sources of phytoestrogens in the Western diet. This study was designed to elucidate whether long term systemic administration of an isoflavone-rich diet is capable of preventing the rapid bone loss occurring after surgical castration in young female rats and, if it is, to gain insight into the mechanisms of this effect.

## Materials and methods

The study was designed to elucidate whether the ingestion of an isoflavone-rich diet before ovariectomy can prevent bone loss following ovariectomy. The experimental rat isoflavone diet was a kind gift from King Car Food Industrial Co., Ltd (Taipei, Taiwan, ROC). Thirty-day-old nulliparous Wistar rats (Animal Center of National Taiwan University Hospital) weighing 180 ± 10 g were housed individually and adapted to a casein-based diet consisting of a nutritionally complete formula [22], including 0.6% calcium, 0.8% phosphorus, and 45 ng of vitamin D3 per gram dry diet. Access to food and water was ad libitum. For each rat, food intake was recorded daily and body weight was recorded weekly throughout the study. Following 1 week of adaptation, 24 rats were randomly divided into four groups. Groups A and B, the normal diet groups, were provided a casein-based diet, consisting of a nutritionally complete formula, throughout the entire three month study period. In Group A, a sham operation without ovariectomy was performed one month after beginning a normal diet (i.e., nutritionally complete formula as above), while the rats in Group B underwent bilateral ovariectomy via a bilateral retroperitoneal approach. The rats of Groups C and D were fed an isoflavone-rich diet (i.e., isoflavone extract from soy beans, 25 g/rat/day) for one month, then bilateral ovariectomy was performed. The diet of the rats in Group C was changed to a normal diet after ovariectomy. The rats in Group D were continuously fed with an isoflavone-rich diet for an additional two months (Table 1).

**Table 1:** Experimental Design (n = 6)

Group	Isoflavone-rich diet 1^st ^month	Isoflavone-rich diet 2^nd ^& 3^rd ^months	Ovariectomy
A	-	-	-
B	-	-	+
C	+	-	+
D	+	+	+

Under general anesthesia with ketamine, ovariectomy was performed by ligation and excision of the ovaries along the upper horns. In the sham operation, the ovaries were exposed as above and gently manipulated, but not excised. In an unpublished pilot study, we demonstrated that bone loss occurs three weeks after ovariectomy. In the early morning after a 12 hour fast, the isoflavones were administered via a funnel-shaped feeding tube through the mouth once a day. During the daytime, the animals were fed Purina Laboratory Chow ad libitum and housed in a temperature-, humidity-, and light-controlled environment. Surgical procedures and experimental protocols were approved and under the supervision of the Animal Research Committee of the National Taiwan University. The rats were sacrificed 60 days after surgery by methofane injection and exsanguination (i.e., on day 90 of the study). For all rats, whole blood samples were obtained with plastic syringes via intracardiac puncture both before surgery and immediate after death. The blood samples were put on ice, centrifuged to separate the serum, divided into aliquots of 500 μl, and deep-frozen at -80°C until further analysis.

After sacrificing the rats, the long bones of the hindlimb, including the femur and tibia, and the thoracolumbar spine, were harvested promptly. The long bones of the right hindlimb and the whole lumbar vertebrae, were trimmed of soft tissue and burned; the weight of bone ash of each bone was then measured for further analysis. A histological study of cancellous bone was done on the proximal femur, the proximal tibia of the left limb, and the last thoracic vertebrae. After necropsy, the last thoracic vertebrae and long bones were dissected out and fixed with 4% formaldehyde in phosphate-buffered solution for 18 hours, followed by decalcification, and then dehydrated in alcohol, cleared in xylene, and embedded in paraffin. Sections, 5 to 7 μm in thickness, were cut and stained with hematoxylin and eosin. Representative sections were photographed using light microscopy. The thickness and interconnections between trabeculae were recorded. The histological observation of bone tissue was performed by a MICD image analyzing system (MICD Software Series, Image Research Inc., Ontario, Canada). The mean percent of porosity of bone tissue was measured by dividing the observed area, without bone tissue, by the whole area.

Biochemical indices of skeletal metabolism, such as alkaline phosphatase (ALP; procedure no. ALP-10, Sigma Co., St. Louis, MO, USA), aspartate aminotransferase, and alanine aminotransferase (AST/GOT and ALT/GPT; procedure no. 505-OP, Sigma Co.), amylase (AMY; procedure no. Amylase-10, Sigma Co.), creatinine (CRE; procedure no. 555-A, Sigma Co.), calcium (CA; procedure no. 587-100P, Sigma Co.), and inorganic phosphorus (IP; procedure no. 363-3-100P, Sigma Co.) content in the serum were measured with commercially available assay kits. Parathyroid hormone (PTH) content in the serum was also measured with commercially available assay kits (IMMULITE 2000 Intact PTH, Diagnostic Products Corporation, Los Angeles, CA, USA).

### Statistical Analysis

The data obtained were evaluated by analysis of variance (ANOVA). Post hoc tests were performed with Bonferroni's test. Statistical significance was considered as a P < 0.05.

## Results

**Figure 1:**
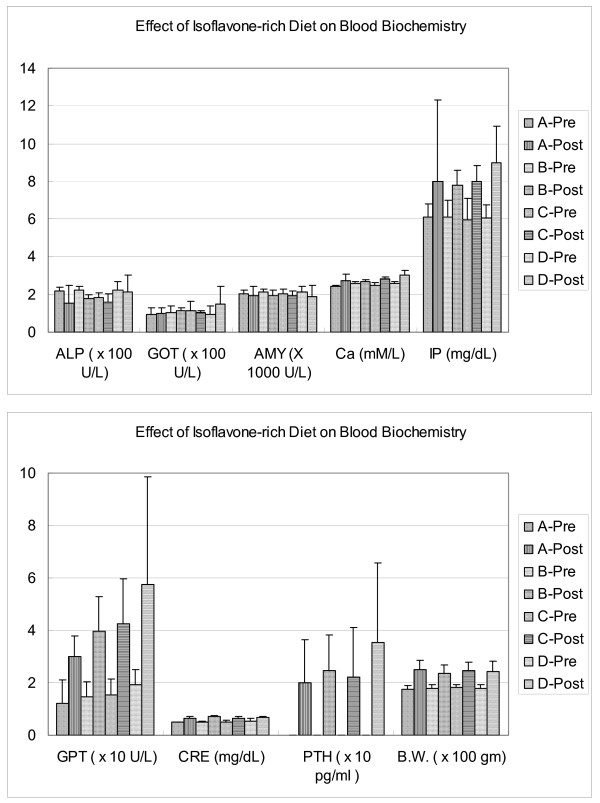
**Biochemical parameters of metabolism after ovariectomy with and without an isoflavone-rich soy protein isolate diet**. After ingestion of an isoflavone-rich diet for one to three months, the biochemical parameters in serum, including alkaline phosphatase, amylase, calcium, GOT (aspartate aminotransferase), and inorganic phosphorus did not show any significant changes (P > 0.05). At the end of the study period, the measurement of body weight, serum creatinine, GPT (alanine aminotransferase), and parathyroid hormone attained a higher value as compared with the preoperative baseline values; however, there was no statistically significant difference between the groups at the end of the study (P > 0.05). Note: A-Pre: Group A rats pre-study; A-Post: Group A rats post-study. B-Pre: Group B rats pre-study; B-Post: Group B rats post-study. C-Pre: Group C rats pre-study; C-Post: Group C rats post-study. D-Pre: Group D rats pre-study; D-Post: Group D rats post-study.

After ingestion of an isoflavone-rich diet for one to three months, the biochemical parameters in serum, including ALP, amylase, CA, GOT, and IP did not change significantly (P > 0.05; Figure 1). At the conclusion of the study, the measurements of body weight, serum creatinine, GPT, and PTH attained a higher value as compared with the preoperative baseline values; however, there were no statistically significant differences between the groups at the end of the study period (Figure 1).

**Figure 2:**
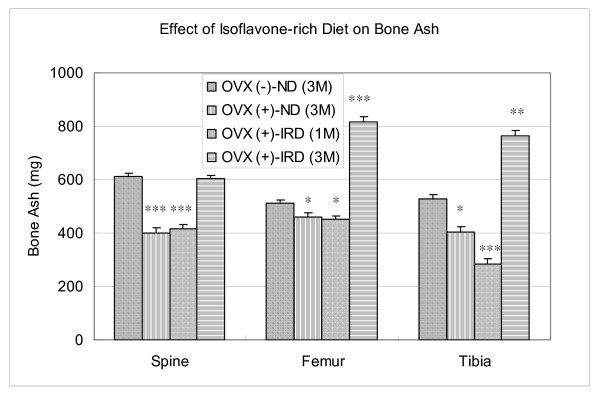
**Weight of long bone ash after ovariectomy with and without an isoflavone-rich diet treatment**. After ovariectomy, the bone ash of long bones in the hindlimbs decreased significantly. When compared with the sham-operated rats fed a normal diet (Group A), ovariectomy (Group B) decreased the bone mass 34.9%, 9.7%, and 23.8% in the spine, femur, and tibia, respectively. Pre-ovariectomy ingestion of an isoflavone-rich diet for one month followed by ovariectomy and a normal diet (Group C) decreased the bone ash, 32.3%, 11.7%, and 45.9% in the spine, femur, and tibia, respectively, while ingestion of an isoflavone-rich diet for three months (Group D), increased the bone ash of the femur and tibia 59.7% and 44.2%, respectively, measured two months after ovariectomy. Note: OVX (-)-ND (3M): Group A, sham-operated rats with a normal diet for three months. OVX (+)-ND (3M): Group B, ovariectomized rats with a normal diet for three months. OVX (+)-IRD (1M): Group C, ovariectomized rats pretreated with an isoflavone-rich diet for one month followed by a normal diet for two months after ovariectomy. OVX (+)-IRD (3M): Group D, ovariectomized rats pretreated with an isoflavone-rich diet for one month followed by an isoflavone-rich diet for an additional two months after ovariectomy. *, P < 0.05; **, P < 0.005 when compared with the non-ovariectomy group [OVX (-)-ND (3M)].

After ovariectomy, the bone ash of long bones in the hindlimbs was significantly lower than that of sham-operated groups (Figure 2). When compared with the sham-operated rats fed a normal diet (Group A), the bone mass of the ovariectomy group (Group B) were 34.9%, 9.7%, and 23.8% lower in the spine, femur, and tibia, respectively. The bone ash of Group C (an isoflavone-rich diet for one month followed by ovariectomy and a normal diet) were 32.3%, 11.7%, and 45.9% lower in the spine, femur, and tibia, respectively; while in Group D (an isoflavone-rich diet for three months), the bone ash of the femur and tibia were 59.7% and 44.2% higher than that of sham-operated normal control rats, respectively (Figure 2).

**Figure 3:**
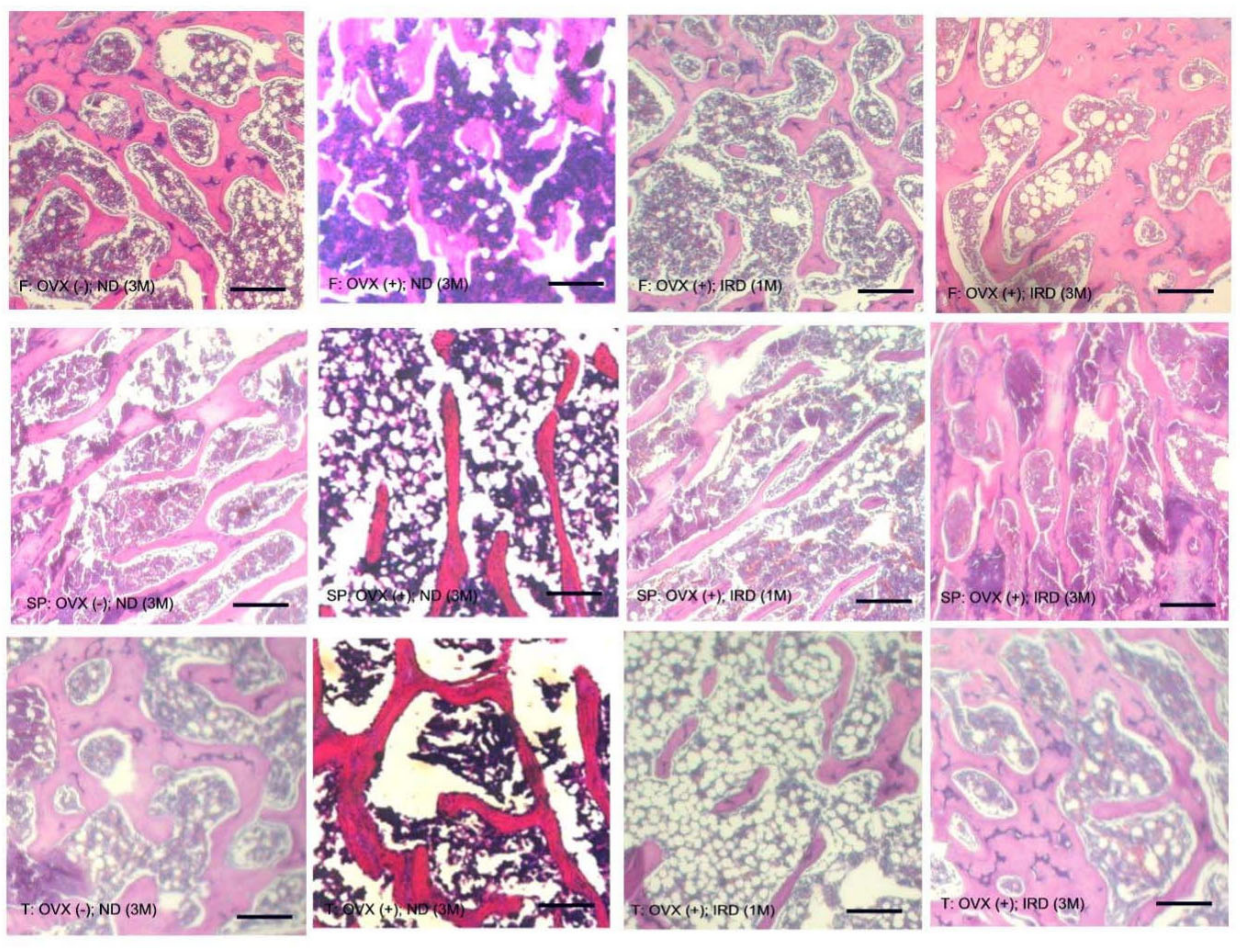
**Histomorphological study of bone trabeculae after ovariectomy and treatment with and without an isoflavone-rich diet**. In the histomorphological study of sham-operated normal rats, the cancellous bone showed intervening trabecular bone with connectivity of the trabecular elements. In rats fed a normal diet throughout the entire study period [(OVX (+)-ND (3M)], there was significant thinning and disconnection of trabeculae after ovariectomy. Rats treated with an isoflavone-rich diet for one month followed by a normal diet after ovariectomy [(OVX (+)-IRD (1M)]. When the rats were fed an isoflavone-rich diet for three months [OVX (+)-SD (3M)], the trabeculation in the cancellous bone of the proximal femur and tibia was significantly higher than that of normal controls. The lumbar vertebrae showed thickening of trabeculae with restoration of interconnections. F: proximal femur; Sp: thoracic spine; T: proximal tibia. OVX (-)-ND (3M): Group A, sham-operated rats with a normal diet for three months. OVX (+)-ND (3M): Group B, ovariectomized rats with a normal diet for three months. OVX (+)-SD (1M): Group C, ovariectomized rats pretreated with an isoflavone-rich diet for one month followed by a normal diet for two months after ovariectomy. OVX (+)-SD (3M): Group D, ovariectomized rats pretreated with an isoflavone-rich diet for one month followed by an isoflavone-rich diet for an additional two months after ovariectomy.

In the histological study of the last thoracic vertebrae in sham-operated normal rats, the cancellous bone showed intervening trabecular bone with connectivity of the trabecular elements. In rats received ovariectomy fed a normal diet throughout the entire study period (Group B), the thinning and disconnection of trabeculae was easily observed as compared with sham-operated normal controls and the decrease in bone trabeculae was most significant in the proximal tibia (Figure 3). In ovariectomized rats provided an isoflavone rich diet for one month followed by a normal diet (Group C), the same histological changes occurred as observed in Group B (Figure 3). When the rats were fed an isoflavone-rich diet for three months (Group D), the trabeculation in the cancellous bone of the proximal femur and tibia was significant higher than that of normal controls. The lumbar vertebrae appeared to show thickening of trabeculae with restoration of interconnections (Figure 3).

### Mean Percent of Porosity

**Figure 4:**
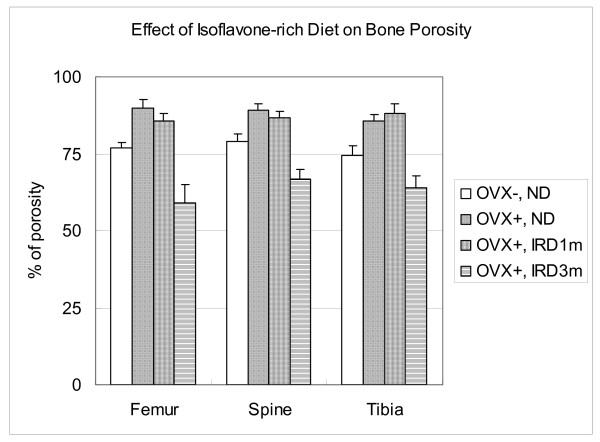
**Mean bone porosity after ovariectomy and treatment with and without isoflavone-rich diet**. In the normal rats (Group A), the mean bone porosity was 76.7%, 79.1%, and 74.3% for the femur, spine, and tibia, respectively. After ovariectomy (Group B), the mean bone porosities significantly increased to 89.8%, 89.2%, and 85.7% for the femur, spine, and tibia, respectively (P < 0.05). The bone porosities of group C (an isoflavone-rich diet for one month followed by ovariectomy and a normal diet) significantly increased to 85.8%, 86.9%, and 88.2% in the femur, spine, and tibia, repectively, while in Group D (an isoflavone-rich diet for three months), the bone porosities of the femur, spine, and tibia decreased to 59.3%, 66.8%, and 64.0%, respectively, two months after ovariectomy (P < 0.05). Ovariectomy increases, while an isoflavone-rich diet decreases bone porosity.

The ovariectomized rats had a higher, while the rats fed with isoflavone-rich diet had a lower bone porosity. In the normal rat (Group A), the mean bone porosity was 76.7%, 79.1%, and 74.3% for the femur, spine, and tibia, respectively. After ovariectomy (Group B), the mean bone porosity were significantly higher with the bone porosity of 89.8%, 89.2%, and 85.7% for the femur, spine, and tibia, respectively (Figure 4, P < 0.05). The bone porosity of Group C (an isoflavone-rich diet for one month followed by ovariectomy and a normal diet) was still isignificantly higher than that of sham-operated normal control with the bone porosity of 85.8%, 86.9%, and 88.2% in the femur, spine, and tibia, respectively. While in Group D (an isoflavone-rich diet for three months), the bone porosities of the femur, spine, and tibia were 59.3%, 66.8%, and 64.0%, respectively, measured two months after ovariectomy; which were significantly lower than that of sham-operated normal control (Figure 4, P < 0.05).

## Discussion

Osteoporosis is diagnosed by its relatively very low bone mineral density (BMD). Moreover, many women who are osteopenic eventually develop osteoporotic fractures [23]. The adult ovariectomized rat is a convenient and reliable experimental model for the evaluation of agents intended to prevent and/or treat postmenopausal osteoporosis. Like humans, rats have cancellous bone that undergoes remodeling once longitudinal growth has essentially ceased [24]. In adult rats, ovariectomy is followed by an increase in bone turnover associated with bone loss and a permanent deficit of bone mass at several skeletal sites, including the vertebral bodies, the proximal femur, and the metaphyses of long bones, such as the distal femur and proximal tibia [24]. In the current study, the decrease in bone mass after ovariectomy was observed in the femur, tibia, and spine, but it was more evident in the metaphysis of the long bones (Figures 2 and 4).

After treatment with an isoflavone-rich diet for three months, most of the biochemical parameters in serum did not show any significant changes. Only the serum titers of CRE, GPT, and PTH were higher than that of the preoperative baseline values, but there were no statistically significant differences between the groups at the end of the study (Figure 1). This observation can be partially explained by the younger group of premenopausal rats with higher endogenous estrogen and active physiologic bone formation at this age [25] or possibly there was no obvious detrimental physiological effect of an isoflavone-rich diet on this particular animal model.

When compared with the sham-operated rats fed a normal diet (Group A), the bone mass of rats with a normal diet after ovariectomy (Group B) decreased significantly. Pre-ovariectomy ingestion of an isoflavone-rich diet for one month did not prevent rats from bone loss (Group C). As noted in this study, the bone ashes analysis revealed a greater than 40% reduction in whole tibial bone mass, greater than a 30% reduction in whole spine bone mass, and greater than a 10% reduction in whole femur bone mass two months following ovariectomy (Figure 2). Following ovariectomy, the microarchitectural alterations in cancellous bone were similar to those observed in postmenopausal and age-dependent osteoporosis, which produced architectural discontinuities of cancellous bone (Figure 3). The lack of an effect of pretreatment with an isoflavone-rich diet in ovariectomized rats is striking, in contrast to its effects on the bone of the ovariectomized animals fed an isoflavone-rich diet for three months. An explanation of this lack of effect is not apparent. Reduction of bone mass in the ovariectomized rats pretreated with an isoflavone rich-diet suggests that premenopausal isoflavone supplementation cannot prevent bone loss and osteoporosis following the menopause. Further, in this rat ovariectomy model, the different degree of bone loss reflects the fact that the early postovariectomy period is characterized primarily by loss of bone mass in the long bones such as the tibia and femur, rather than in the spine.

In this study, the rats were young adults and had not yet achieved the slowest phase of growth. We found that although prolonged isoflavone-rich diet intake has a positive effect on the bone mass, the cessation of an isoflavone-rich diet intake does not guarantee that the bone-sparing effect will persist. This raises the question whether the presence of a well-formed and active growth plate may have affected the action of an isoflavone-rich diet on bone. This fact can be partially explained by the use of growing animals, as the remodeling of the primary spongiosa is also suppressed and the increase in bone area is not only due to a stimulation of growth but also may be due to the failure to remodel the primary spongiosa. Studies in older rats, or ideally in another experimental model that has a true Haversian system and with remodeling cancellous bone, would help to answer this question.

The bone mass of the rats with ingestion of an isoflavone-rich soy protein isolate for three months still higher than that of the sham-operated normal control even at two months after ovariectomy. The treatment of rats with an isoflavone-rich diet for three months resulted in an approximately 40–60 % increase of bone mass in the long bones even when measured two months after ovariectomy (Figure 2). These findings are consistent with the recent report of Arjmandi BH et al. (1996) that dietary intake of soybean products prevents bone loss after ovariectomy [26]. In a human study, Mei et al. demonstrated that a habitually high intake of dietary isoflavone is associated with a higher bone mineral density value in both the spine and hip regions, while no association existed between dietary phytoestrogen intake and bone mineral density value in premenopausal women with high endogenous estrogen [25]. In this study, because there were no bone ash and histology or histomorphometry measurement on animals after one month feeding isoflavones, we can not concluded that habitual ingestion of an insoflavone-rich diet during the premenopause had a protective effect against bone loss after ovariectomy. The bone-sparing action did observed in the treated group with long-term ingestion of an isoflavone-rich diet. The bone-sparing action of isoflavone supplementation primarily effect the metaphyseal portion of the proximal tibia and the vertebrae of spine. Customarily, high isoflavone intake may also help to reverse the state of secondary hyperparathyroidism associated with estrogen withdrawal and hence lower the rate of bone turnover in postmenopausal women [25]. In our study, the rats fed with an isoflavone-rich diet were protected completely from bone loss after castration, which is similar to that reported by Arjmandi et al. [26]. Whether the discrepancy between our study and that of Arjmandi et al. [26] is that bone mass was increased after intake of an isoflavone-rich diet for three months need to be further validated in the future study.

The possibility that the effect of 25 mg/rat/day of isoflavones on bony tissues of the ovariectopmized rats is via a non-estrogenic mechanism is supported by the presented noninvasive and histological results. In fact, the higher number of bone tracbeculae measured in the ovariectomized- rats fed an isoflavone-rich soy protein isolate is indicative of a preventive effect of isoflavones on bone loss (Figure 3). This response of bone is different from that expected following administration of estradiol, since most investigators have reported that the rate of bone formation of ovariectopmized rats is suppressed, rather than stimulated, by exogenous estradiol [27-29]. Whether the preventive effect on bone loss of an isoflavone-rich diet on bone formation is mediated by a preferential interaction of this ligand with the estrogen receptor β, or by interaction with selective estrogen receptor modulators such as the raloxifene-response element, or by non-receptor-mediated direct effects on specific intracellular enzymes, warrants future investigation.

In summary, there are limitations in this study. The first one is the animal age, which is much younger than that usually used for the postmenopusal model; the second one is that the bone histological study used in this study is mainly a 2-dimensional approach with inherent model-based assumption, i.e., indirect measurement of microstructure; and the housing of rats individually may be also a stressor that confounds interpretation [30]. However, the premenopausal ingestion of isoflavone does not prevent the development of post-ovariectomized bone loss, while the habitual ingestion of an isoflavone-rich diet does increase the bone mineral contents in the long bones of ovariectomized rats. The mechanism by which an isoflavone-rich soy protein isolate acts on bone resorption is still unknown and should be the subject of additional investigation.
